# Variation between surgeons in rate of reoperation after horizontal strabismus surgery among Medicare beneficiaries: associations with patient and surgeon characteristics and adjustable sutures

**DOI:** 10.22336/rjo.2024.03

**Published:** 2024

**Authors:** Christopher T. Leffler, Alicia Woock, Meagan Shinbashi, Melissa Suggs

**Affiliations:** * Department of Ophthalmology, Virginia Commonwealth University, Richmond, Virginia, U.S.A.; ** Department of Ophthalmology, Richmond VA Medical Center, Richmond, Virginia, U.S.A.; *** Department of Ophthalmology and Visual Sciences, School of Medicine, University of Alabama at Birmingham, Birmingham, Alabama, U.S.A.; **** OHSU Casey Eye Institute, Oregon Health & Science University, Portland, Oregon, U.S.A.

**Keywords:** surgery, reoperation, strabismus, variation

## Abstract

**Objective:** To quantify variation between surgeons in reoperation rates after horizontal strabismus surgery, and to explore associations of reoperation rate with surgical techniques, patient characteristics, and practice type and volume.

**Methods:** Fee-for-service payments in a national database to providers for Medicare beneficiaries having strabismus surgery on horizontal muscles between 2012 and 2020 were analyzed retrospectively to identify same calendar year reoperations. Multivariable linear regression was used to determine predictors of each surgeon’s reoperation rate.

**Results:** The reoperation rate for 1-horizontal muscle surgery varied between 0.0% and 30.8% among 141 surgeons. Just 7.8% of surgeons contributed over half of the reoperation events for 1-horizontal muscle surgery, due to the presence of high-volume surgeons with high reoperation rates. Surgeon seniority, gender, surgery volume, and use of adjustable sutures were not independently associated with surgeon reoperation rate. We explored associations of reoperation with patient characteristics, such as age and poverty. Surgeons in the South tended to have a higher reoperation rate (p=0.03) in a multivariable model. However, the multivariable model could only explain 16.3% of the inter-surgeon variation in reoperation rate for 1-horizontal muscle surgery.

**Discussion:** Strabismus surgery is similar to other areas of medicine, in which large variations in outcomes between surgeons are observed. Future work can be directed towards explaining this variation.

**Conclusions:** Patient-level analyses that fail to consider variation between surgeons will be dominated by a small number of high-reoperation, high-volume surgeons. Order-of-magnitude variations exist in reoperation rates among strabismus surgeons, the cause of which is largely unexplained.

## Introduction

Inter-surgeon variation in patient outcomes has been explored for non-ophthalmic procedures [**1**,**2**] as well as ophthalmic surgeries, such as cataract extraction [**3**,**4**] and corneal transplantation [**5**]. We sought to study inter-surgeon variation in strabismus surgery outcomes. Reoperation rate is one frequently used outcome metric for strabismus surgery [**6**-**12**]. We hoped to determine if inter-surgeon variation in reoperation rate after strabismus surgery could be explained: 1) by surgical approach, such as adjustable suture use; 2) by characteristics of the surgeon, such as seniority, gender, or practice volume; or 3) by aspects of the practice’s patient population, such as poverty or age.

We evaluated strabismus surgery reoperations in the database of payments from Medicare to providers from 2012 to 2020 [**13**].

## Methods

This study was approved by the Office of Research Subjects Protection at our hospital. We used the United States database of Medicare payments from 2012 to 2020 [**13**]. This database contained data on payments for every practitioner in the United States who received Medicare fee-for-service payments. Medicare is a national, single-payer health insurance program administered by the United States government, which serves patients aged 65 or over, and other patients who have disabilities. Each current procedural terminology (CPT) code must be paid to a provider for at least 11 beneficiaries in a single year for the CPT to be listed for the year, for that provider. We also downloaded characteristics of patient demographic and clinical information for each provider’s Medicare practice, for the mid-point year (2016), or, if data were not available for the mid-point year for a provider, whichever year was closest to this year [**14**].

We defined senior surgeons as those who left the Medicare database during the 2012 to 2020 period, junior surgeons as those who entered the Medicare database during this period, and other surgeons, as mid-career.

We evaluated the reimbursed reoperation rate in patients having strabismus surgery for one horizontal muscle (CPT 67311). Findings were analyzed concerning whether practices coded for adjustable suture placement (CPT 67335), one vertical muscle surgery (CPT 67314), and surgery with restrictive myopathy (e.g., thyroid ophthalmopathy) or scarring of extraocular muscles (e.g., prior retinal detachment or strabismus surgery, or prior ocular injury, CPT 67332). The reoperation rate for each surgeon was determined by the number of beneficiary service days and beneficiaries. For instance, if in a given year, a given provider treated 14 beneficiaries with a certain CPT code, but there were 15 beneficiary service days for the code, then a reoperation occurred for 1 of the 14 beneficiaries. The surgeon was the unit of analysis. If payments for CPT 67335 were received by the surgeon, then the adjustable-suture technique was available to the provider.

We evaluated associations of reoperation rate with surgery in a practice with the lowest or highest quartile of surgical volume, and with academic or community practice. Reoperation rate was evaluated in major geographic regions - West (WA, HI, CA, AK, WY, NV, UT, MT, NM, ID, CO, AZ), Midwest (SD, ND, NE, MO, MN, KA, IA WI, OH, MI, IL, IN), Northeast (PA, NY, NJ, VT, RI, NH, MA, ME, CT), and South (TX, OK, LA, AR, TN, MS, KY, AL, WV, VA SC, NC, GA, FL, DC, MD, DE) [**15**]. We excluded retinal oncologist data, as they could have been coding for muscle surgeries when detaching muscles for placement of radiotherapy plaques. 

A t-test was used to compare reoperation rates. Median population values for the patients in a practice were used for group practices. Multivariable linear regression was used to analyze variables found to be significant in univariate analysis. Because of clinical interest in the question, adjustable suture availability was included in the model.

## Results

**Fig. 1:**
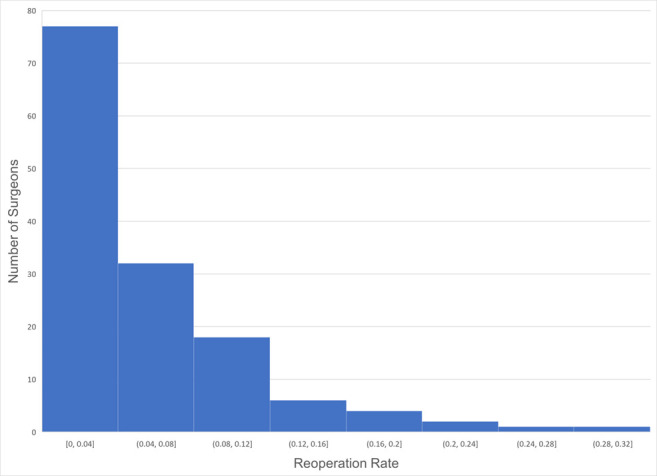
Histogram of reoperation rate for 141 surgeons for surgery on one-horizontal muscle (CPT 67311)

Among 141 surgeons coding for surgery on one-horizontal muscle (CPT 67311), the mean reoperation rate was 4.93% (SD 5.77%), and the median value was 3.57%. However, the range observed was wide, from 0 to 30.8% (**Fig. 1**).

Mean patient age in the practice of at least 71 years was associated with a higher reoperation rate (5.68% vs. 3.65%, p=0.03, **Table 1**. In addition, a prevalence of Medicaid qualification (a marker of poverty) at or above the median value (15.17%) was associated with a lower reoperation rate (3.71% vs. 6.33%, p=0.01, **Table 1**). Southern surgeons had a higher reoperation rate (6.27% vs. 3.94%, p=0.03, **Table 1**).

**Table 1:** Reoperation rate after strabismus surgery on one-horizontal muscle (Current Procedural Terminology 67311) by physician

	Reoperation rate, Mean % (SD %, n), when Factor:		p-value
	Present	Absent	
Surgeon factors:			
Peds/strabismus	4.74% (5.40, 125)	6.44% (8.21, 16)	0.43
Neuro-eye	7.00% (8.65, 14)	4.71% (5.37, 127)	0.35
Oculoplastics	2.53% (1.59, 2)	--	--
Junior	3.21% (2.91, 13)	5.11% (5.97, 128)	0.06
Mid-career	5.10% (5.71, 121)	--	--
Senior	5.20% (10.09, 7)	4.92% (5.52, 134)	0.94
Academic	4.70% (4.83, 54)	5.08% (6.31, 87)	0.69
South	6.27% (7.30, 60)	3.94% (4.09, 81)	0.03
West	3.68% (4.11, 35)	5.35% (6.19, 106)	0.07
Female surgeon	5.31% (5.68, 34)	4.88% (5.90, 107)	0.71
Volume ≥ 37 pts.	5.14% (4.37, 71)	4.72% (6.94, 70)	0.67
Early beneficiaries 2012-16 ≥ 50% of total (2012-20)	5.98% (7.09, 70)	3.90% (3.87, 71)	0.03
Other CPT codes used…			
Adjustable suture (67335)	5.18% (6.54, 46)	4.82% (5.40, 95)	0.75
2-muscle horizontal surgery (67312)	7.78% (5.47, 18)	4.52% (5.72, 123)	0.03
1-muscle vertical surgery (67314)	5.59% (5.45, 65)	4.37% (6.02, 76)	0.21
Scarring/reoperation (67332)	6.05% (4.49, 40)	4.49% (6.17, 101)	0.10
Patient characteristics:			
Mean age ≥ 71 years	5.68% (6.16, 89)	3.65% (4.85, 52)	0.03
Female ≥ 57.23%	5.34% (6.39, 71)	4.52% (5.09, 70)	0.40
Medicaid ≥ 15.17%	3.71% (4.58, 63)	6.33% (6.71, 63)	0.01
White race ≥ 86.62%	6.70% (6.70, 51)	4.17% (5.41, 50)	0.04
Diabetes ≥ 25.0%	5.30% (6.88, 71)	4.89% (4.43, 60)	0.68
Stroke ≥ 8.0%	6.37% (6.63, 41)	4.46% (6.27, 38)	0.19
CMS-HCC Risk score ≥ 1.16	5.58% (6.40, 71)	4.28% (5.02, 70)	0.18
All practices	4.93% (5.77, 141)	--	--
Median values for each practice for the patient characteristics were a volume of 37 patients total from 2012 to 2020; Medicaid qualification (an indicator of poverty) of 15.17%; white race of 86.62%, and fraction with diabetes of 25.0%. The median CMS Hierarchical Condition Category (HCC) risk score for the practices was 1.1604.			

Some findings suggested that strabismus etiology impacted the reoperation rate, though the findings were not generally statistically significant. Surgeons who coded for 2 horizontal muscles in 1 eye (CPT 67312) had a higher reoperation rate (7.78% vs. 4.52%, p=0.03, **Table 1**). Neuro-ophthalmologists tended to have a high reoperation rate (7.00% vs. 4.71%, p=0.35), as did practices with a prevalence of stroke above the median value (reoperation rate 6.37% vs. 4.46%, p=0.19), but the findings were not statistically significant (**Table 1**). 

**Fig. 2:**
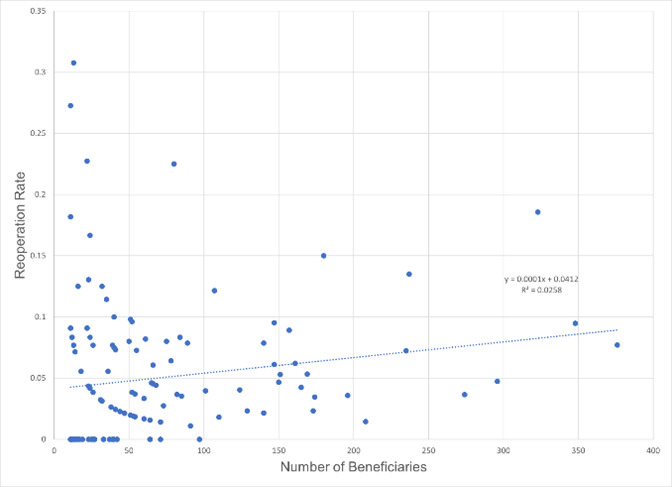
Funnel plot of reoperation rate in the same calendar year as a function of surgical volume for surgery on one horizontal muscle (CPT 67311) for 141 surgeons from 2012 to 2020

Inexperience was not demonstrated to be associated with an elevated reoperation rate. The reoperation rate was not higher for practices (rate 4.72%) with a volume below the median value (37 patients from 2012 to 2020), compared to other practices (rate 5.14%, p=0.67, **Table 1**, **Fig. 2**). Also, junior surgeons (rate 3.21%) did not have a reoperation rate above that of other surgeons (5.11%, p=0.06, **Table 1**).

**Fig. 3:**
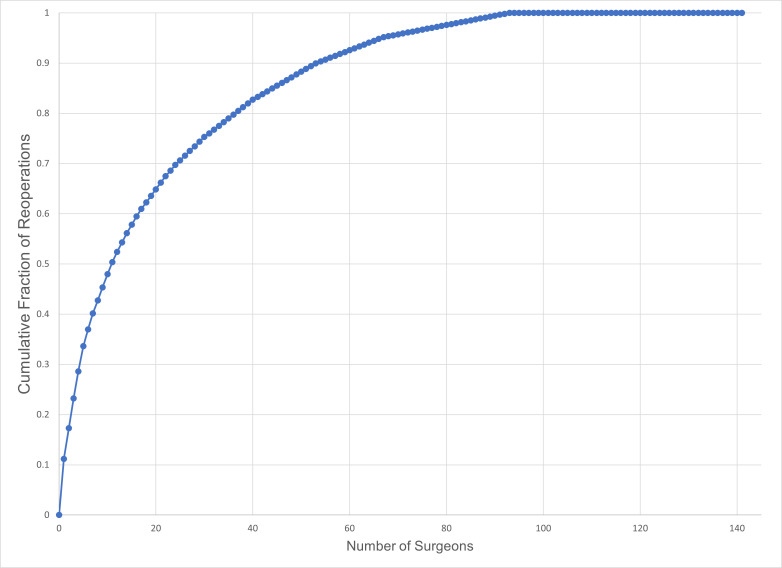
Cumulative contribution of reoperations to the dataset for muscle surgery for one horizontal muscle (CPT 67311) for 141 surgeons

Due to high-volume, high-reoperation surgeons, a low number of surgeons contributed a substantial fraction of the reoperations in this dataset. Just 7.8% of the 141 surgeons coding for CPT 67311 contributed 50.4% of the total number of reoperations in this dataset (**Fig. 3**). Among these high-influence surgeons, adjustable sutures were used by 27%. 

The 46 surgeons who received adjustable suture reimbursement (CPT 67335) had a reoperation rate that was slightly higher than that of the 95 surgeons who did not (5.18% vs. 4.82%, p=0.75, **Table 1**). Similarly, in the multivariable analysis, adjustable suture availability was associated with a nonsignificant elevation in reoperation rate of 0.75% (p=0.57, **Table 2**).

**Table 2:** Multivariable prediction of reoperation rate (%) after surgery on one-horizontal muscle (CPT 67311)

	Regression coefficient (95% CI)	p-value
Adjustable sutures used (CPT 67335)	0.75 (-1.87 to 3.38)	0.57
Two-horizontal muscle surgery used (CPT 67312)	1.65 (-1.70 to 5.01)	0.33
Practice in the South	2.73 (0.23 to 5.23)	0.03
Early beneficiaries 2012-16 ≥ 50% of total (2012-20)	1.95 (-0.39 to 4.29)	0.10
Patient population:		
…Mean age ≥ 71 years.	1.06 (-2.10 to 4.23)	0.51
…Medicaid ≥ 15.17%	-1.26 (-4.38 to 1.86)	0.43
…White patients ≥ 86.62%	1.50 (-1.16 to 4.17)	0.26
Intercept	1.74 (-2.53 to 6.02)	0.42
n=101 surgeons analyzed with complete data. Model r2=0.163.		

By multivariable regression, Southern practices had a reoperation rate 2.73% higher (p=0.03). The predominance of beneficiaries from the early years (2012-16) of the dataset (2012-20) was associated with an elevation in the rate of reoperation of 1.96%, which was not significant (p=0.10, **Table 2**). The multivariable analysis explained 16.3% of the variation between surgeons in the reoperation rate. 

## Discussion

This study examined inter-surgeon variation in reoperation rate after horizontal strabismus surgery in older adults.

Paradoxically, we observed that the reoperation rate after strabismus surgery did not obey the reductions that might have been expected with experience. The reoperation rate was not lower for high-volume surgeons or for those more senior in their careers.

A related observation was that high-volume surgeons with elevated reoperation rates tended to dominate any patient-level analyses that ignored differences between surgeons. Just 7.8% of the surgeons contributed over half the reoperations for surgery on one horizontal muscle. Thus, patient-level analyses that ignored the inter-surgeon variation might have been describing the idiosyncratic practice approaches of a handful of surgeons, rather than yielding generalizable knowledge. 

Geographic variation in outcomes from strabismus surgery [**7**,**10**,**11**] and in rates of the procedure [**16**,**17**] have been demonstrated, though with some inconsistencies. Practices with elevated rates of reoperation can introduce spurious results when practice variation is ignored. In our study, each surgeon contributed one observation, and less regional variation was seen, although Southern surgeons had a higher reoperation rate for surgery on one horizontal muscle.

Previous studies have observed that paralytic strabismus is associated with higher reoperation rates [**8**,**12**]. Similarly, our study noted higher reoperation rates for horizontal surgery in univariate analysis in practices that billed for recess-resect procedures (CPT 67312). We also noted nonsignificant tendencies for elevated reoperation rates in practices with a higher prevalence of stroke and among neuro-ophthalmologists.

Older patient age was associated with an elevated reoperation rate in previous studies [**8**,**11**] and in our study. Reoperation rates in the first calendar year for horizontal surgery varied between surgeons, between zero and 30.8%. Despite the many surgeon and patient variables evaluated, most of the variation in reoperation rate between surgeons remained unexplained.

## Conclusions

Patient-level analyses of strabismus surgery reoperations that fail to consider variation between surgeons will be dominated by a small number of high-reoperation, high-volume surgeons. Order-of-magnitude variations exist in reoperation rates among strabismus surgeons, the cause of which is largely unexplained.

**Conflict of Interest Statement**

The authors have no conflicts of interest and no competing interests to disclose. 

**Informed Consent and Human and Animal Rights Statement**

N/A. 

**Authorization for the use of human subjects**

Ethical approval: The research related to human use complies with all the relevant national regulations and institutional policies, is by the tenets of the Helsinki Declaration, and has been approved by the Office of Research Subjects Protection, Virginia Commonwealth University, Richmond, Virginia, U.S.A. (#HM20026313, on Dec. 16, 2022).

**Acknowledgments**

None.

**Sources of Funding**

None.

**Disclosures**

None.

**Presentation**

Presented at the American Ophthalmological Society meeting in Asheville, NC, May 19, 2023.
